# DAB2IP inhibits glucose uptake by modulating HIF-1α ubiquitination under hypoxia in breast cancer

**DOI:** 10.1038/s41389-024-00523-4

**Published:** 2024-06-11

**Authors:** Hongliang Dong, Weiyi Jia, Weijian Meng, Rui Zhang, Zhihong Qi, Zhuo Chen, Sophia Xie, Jiang Min, Liang Liu, Jie Shen

**Affiliations:** 1 Department of GI Surgery, Tongji Hospital, Tongji Medical College, Huazhong University of Science and Technology, Wuhan, 430030 China; 2 GI Cancer Research Institute, Tongji Hospital, Tongji Medical College, Huazhong University of Science and Technology, Wuhan, 430030 China; 3 Department of Science & Education, Zhengzhou Central Hospital Affiliated to Zhengzhou University, Zhengzhou, 450007 China; 4 Wuhan Britain-China School, Wuhan, 430030 China; 5 Gastrointestinal Surgery Department, The First Affiliated Hospital of Chongqing Medical University, Chongqing, 40000 China

**Keywords:** Breast cancer, Cancer metabolism

## Abstract

Metabolic reprogramming has become increasingly important in tumor biology research. The glucose metabolic pathway is a major energy source and is often dysregulated in breast cancer. DAB2IP is widely reported to be a tumor suppressor that acts as a scaffold protein to suppress tumor malignancy in breast cancer. Interestingly, DAB2IP has also been found to be a potential regulator of glucose uptake; however, the exact mechanism remains unclear. In this study, we found that DAB2IP inhibited glucose uptake under hypoxia conditions in breast cancer cells by suppressing HIF-1α signals. Mechanically, DAB2IP interacted with the E3 ubiquitin ligase STUB1 via its PER domain, thus triggering STUB1 mediated HIF-1α ubiquitylation and degradation, and inhibit glucose metabolism and tumor progression. Deleting the PER domain abrogated the DAB2IP-related inhibitory effects on glucose uptake, intracellular ATP production, and lactic acid production in breast cancer cells. These findings elucidate the biological roles of DAB2IP in cancer-related glucose metabolism as well as a novel mechanism by which STUB1-driven HIF-1α ubiquitylated degradation is regulated in breast cancer.

## Introduction

Over the past four decades, the incidence of breast cancer has steadily risen [1]. Metabolic reprogramming during the malignant transformation of breast tumors is a key characteristic of cancer and plays an important role in facilitating tumor cell proliferation [2]. Glucose metabolism, a major energy source, is often dysregulated in breast cancer [2, 3]. Multiple transporters and enzymes reportedly involved in glucose metabolism are highly expressed and associated with prognosis in breast malignancies, including glucose transporter 1–6 and 12 (GLUT1-6, 12) [4], hexokinase 2 (HK2) [5], 6-phosphofructo-2-kinase/fructose-2, 6-biphosphatase 3 (PFKFB3) [6], and pyruvate kinase M2 (PKM2) [7]. Various signals have been reported to be involved in the regulation of GLUTs and glucose-related enzyme expression, such as the PI3K/AKT, AMPK, MAPKs, Wnt, and mTOR pathways [8–12]; however, the concrete upstream mechanism that controls glucose metabolic processes in breast cancer requires further investigation.

DAB2IP, also called ASK1 interacting protein, has been reported to modulate multiple oncogenic pathways and act as a tumor suppressor in multiple tumors [13]. In breast cancer, DAB2IP is aberrantly methylated and inactivated, thus inducing tumor invasion and lymphatic metastasis [14]. DAB2IP is a member of the Ras GTPase-activating protein family and often interacts directly with DAB2 to regulate various biological processes, such as cell proliferation, apoptosis, and metastasis [15]. Our recent research showed that DAB2IP can also act as a scaffold protein to suppress tumor malignancy and invasiveness in a ubiquitylation-dependent manner [16, 17]. While DAB2IP regulates glucose uptake in patients with type 2 diabetes [18], it remains unclear whether DAB2IP affects glucose metabolism in cancer cells.

In this study, DAB2IP was found to inhibit glucose uptake under hypoxic conditions in breast cancer cells by suppressing HIF-1α signals. DAB2IP interacted with the E3 ubiquitin ligase STUB1, thus triggering STUB1-mediated HIF-1α ubiquitylation and degradation, inhibiting glucose metabolism and tumor progression. Furthermore, we found that the PER domain of DAB2IP was essential for STUB1-mediated HIF-1α ubiquitylation and that deleting the PER domain may cancel the DAB2IP-related inhibitory effect of glucose uptake, intracellular ATP production, and lactic acid production in breast cancer. Taken together, our findings highlight the biological role of DAB2IP in cancer-related glucose metabolism and elucidate a novel mechanism of DAB2IP in regulating STUB1-driven HIF-1α ubiquitylated degradation in breast cancer. Moreover, DAB2IP may serve as a potential therapeutic target for disrupting aberrant HIF-1α signaling in tumor-targeted therapy.

## Results

### DAB2IP acted as a tumor suppressor and was involved in glucose metabolism in breast cancer

**Fig. 1:**
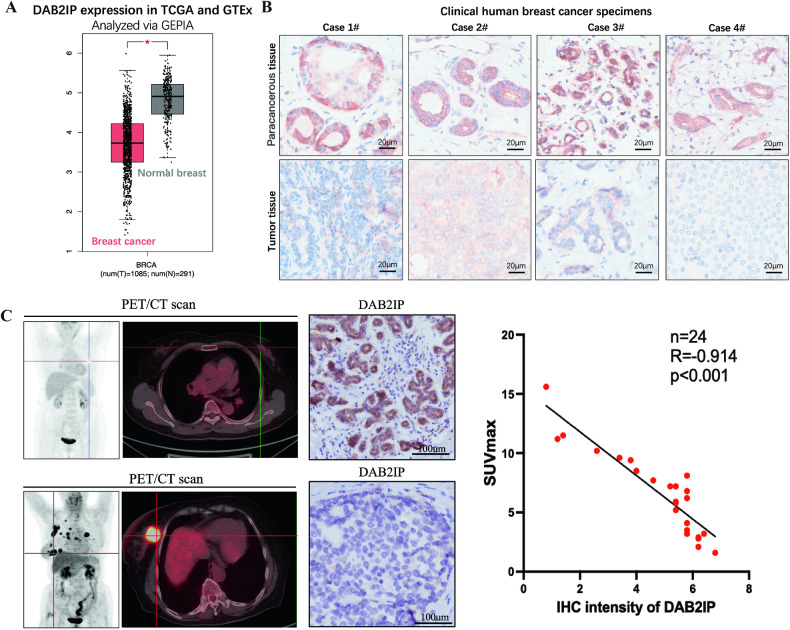
DAB2IP acted as a tumor suppressor and was involved in glucose metabolism in breast cancer. **A** Relative expression level of DAB2IP in public database of breast cancer (*n* = 1085) and normal breast tissue (*n* = 291). Expression profile was obtained from TCGA-BRCA and GTEx databases and analyzed via GEPIA software. *p < 0.05. **B** Immunohistochemistry staining of DAB2IP protein in paired para-cancerous and tumor tissues from four clinical breast cancer patients. Scale bar: 20 μm. **C** 18F-FDG PET/CT scan and DAB2IP immunohistochemistry staining of breast cancer primary tumor lesion (*n* = 24). Glucose uptake level was evaluated via the maximum standard uptake value of 18F-FDG (SUVmax), and the expression level of DAB2IP was presented as immunohistochemistry staining intensity (IHC intensity). Pearson correlation analysis was performed between SUVmax and DAB2IP IHC intensity (*R* = -0.914, *p* < 0.001).

We evaluated the expression levels of DAB2IP in a public database of breast cancer (TCGA-BRCA) and normal breast tissues (GTEx). Compared to normal tissues, DAB2IP gene expression was significantly decreased in breast cancer (Fig. 1A). Similarly, immunohistochemistry (IHC) examination of four paired clinical human breast cancer specimens showed that DAB2IP staining intensity was higher in para-cancerous tissues than in tumor tissues (Fig. 1B). Therefore, we deduced that DAB2IP may act as a potential tumor suppressor in breast cancer. Next, we screened for potential DAB2IP-correlated genes (*p* < 0.05) in TCGA-BRCA using the R2 platform (Supplementary Fig. 1A). Through gene set enrichment analysis, we found that DAB2IP negatively regulates oxidative phosphorylation (Supplementary Fig. 1B), which acts downstream of glucose metabolism.

To further determine whether DAB2IP expression is associated with glucometabolism, we retrospectively collected data from 24 patients with breast cancer who underwent 18F-FDG PET/CT and tumor biopsy. IHC staining and PET/CT showed that the DAB2IP expression level was negatively correlated with 18F-FDG maximum standard uptake value (SUVmax) of the primary tumor (R = -0.914, *p* < 0.001) (Fig. 1C). These results indicated that DAB2IP acts as a tumor suppressor and negatively regulates glucose uptake in breast cancer.

### DAB2IP inhibited glucose uptake of breast cancer cells during hypoxia

**Fig. 2:**
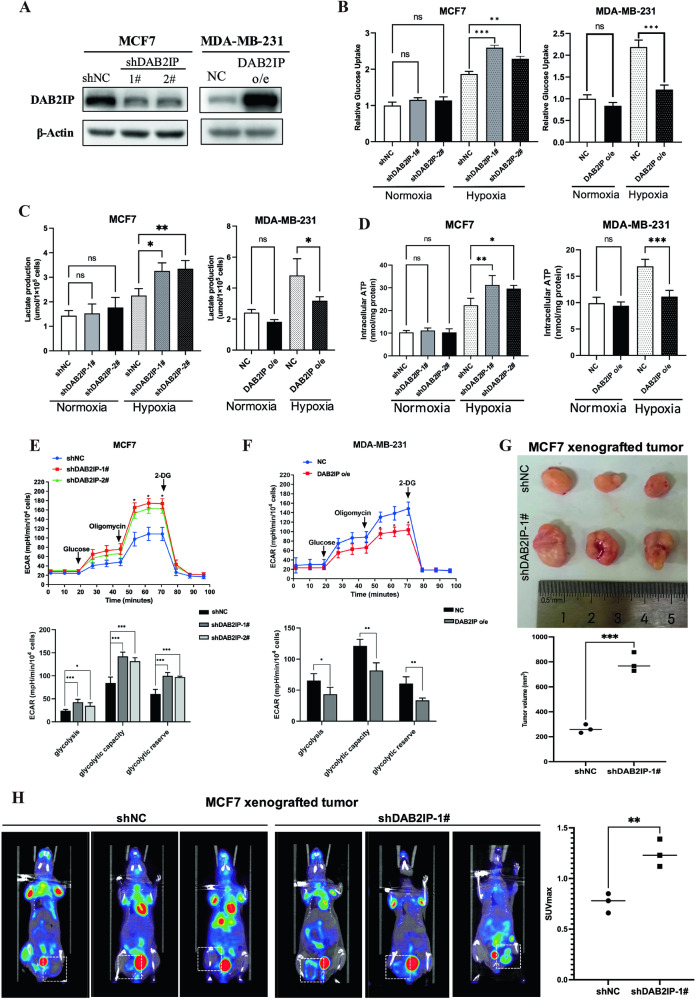
DAB2IP inhibited glucose uptake of breast cancer cells during hypoxia. **A** Western blot verifying knockdown of endogenous DAB2IP via shDAB2IP plasmids in MCF7 cells (shDAB2IP-1#, shDAB2IP-2#) and MDA-MB-231 cells with DAB2IP overexpression (DAB2IP o/e). sh-scramble (shNC) and pcDNA3.1 empty vector (NC) plasmids were used as negative control. **B** Glucose uptake assay was performed in MCF7 cells with DAB2IP knockdown (shDAB2IP-1# and shDAB2IP-2#) and MDA-MB-231 cells with DAB2IP overexpression (DAB2IP o/e) under normoxia or hypoxia condition. sh-scramble (shNC) and pcDNA3.1 empty vector (NC) plasmids were used as negative control. Hypoxia was triggered via anaerobic incubation for at least 8 h. Three bio-replications were performed for each experiment. ns, no significant; **p < 0.01; ***p < 0.001. **C** Lactate production was examined in MCF7 cells with DAB2IP knockdown (shDAB2IP-1# and shDAB2IP-2#) and MDA-MB-231 cells with DAB2IP overexpression (DAB2IP o/e) under normoxia or hypoxia conditions. sh-scramble (shNC) and pcDNA3.1 empty vector (NC) plasmids were used as negative control. Hypoxia was triggered via anaerobic incubation for at least 8 h. Three bio-replications were performed for each experiment. ns, no significant; *p < 0.05; **p < 0.01. **D** Intracellular ATP production was examined in MCF7 cells with DAB2IP knockdown (shDAB2IP-1# and shDAB2IP-2#) and MDA-MB-231 cells with DAB2IP overexpression (DAB2IP o/e) under normoxia or hypoxia condition. sh-scramble (shNC) and pcDNA3.1 empty vector (NC) plasmids were used as negative control. Hypoxia was triggered via anaerobic incubation for at least 8 h. Three bio-replications were performed for each experiment. *p < 0.05; **p < 0.01; ***p < 0.001. **E** Representative ECAR profiles of MCF7 cells transfected with sh-scramble (shNC, blue line), shDAB2IP-1# (red line) and shDAB2IP-2# (green line) following glucose, oligomycin, and 2-DG treatments. Cells were cultured in an anaerobic incubator for at least 8 h before the experiment. ECAR of glycolysis, glycolytic capacity, and glycolytic reverse in MCF7 cells transfected with sh-scramble (shNC) and shDAB2IP (shDAB2IP-1# and 2#). Three bio-replications were performed for each experiment. ECAR, extracellular acidification rate; 2-DG, 2-Deoxy-D-glucose; *p < 0.05; **p < 0.01; ***p < 0.001. **F** Representative ECAR profiles of MDA-MB-231 cells transfected with empty vector (NC, blue line) and DAB2IP overexpression plasmid (DAB2IP o/e, red line) following glucose, oligomycin and 2-DG treatments. Cells were cultured in anaerobic incubator for at least 8 h before experiment. ECAR of glycolysis, glycolytic capacity and glycolytic reverse in MDA-MB-231 cells transfected with empty vector (NC) and DAB2IP overexpression plasmid (DAB2IP o/e). Three bio-replications were performed for each experiment. ECAR, extracellular acidification rate; 2-DG, 2-Deoxy-D-glucose; *p < 0.05; **p < 0.01. **G** Four-week-old female BABL/c nude mice were subcutaneously injected 1 × 10^6^ MCF7 cells with DAB2IP knockdown (shDAB2IP-1#) or control (shNC) (*n* = 3 per group). After 4 weeks, micro-PET/CT scan was performed, then tumors were removed and measured. ***p < 0.001. **H** 18F-FDG micro-PET/CT scan for MCF7 xenografted tumors with DAB2IP knockdown (shDAB2IP-1#) or control (shNC) from Fig. 2G. Glucose uptake level was evaluated via the maximum standard uptake value of 18F-FDG (SUVmax). **p < 0.01.

To evaluate the role of DAB2IP in glucose metabolism, we examined its expression in six breast cancer cell lines. Western blot showed that DAB2IP was highly expressed in MCF7, T47D, and ZR-75-1 cells and was relatively abrogated in MDA-MB-231, MDA-MB-468, and HCC1937 (Supplementary Fig. 1C). MDA-MB-231 and MCF7 cells have been reported to have relatively high and low tumor aggressiveness, respectively [19]; thus, these two cell lines were selected for further study. The stable knockdown and overexpression of DAB2IP in MCF7 and MDA-MB-231 cells, respectively, were validated by western blotting (Fig. 2A). Glucose and lactate detection assays revealed that DAB2IP only inhibited glucose uptake and lactate production in MCF7 and MDA-MB-231 cells under hypoxic conditions but not under normoxic conditions (Fig. 2B, C). DAB2IP also inhibited the intracellular ATP production in both MCF7 and MDA-MB-231 cells under hypoxic conditions (Fig. 2D). Similarly, DAB2IP could more significantly inhibit cell viability in MDA-MB-231 and MCF7 under hypoxia (Supplementary Fig. 1D, E). Additionally, the Seahorse system was used to evaluate the real-time extracellular acidification rate (ECAR) in both MCF7 and MDA-MB-231 cells. The results showed that DAB2IP knockdown significantly increased the ECAR of basal glycolysis and glycolytic capacity in MCF7 cells (Fig. 2E), whereas DAB2IP overexpression decreased the ECAR in MDA-MB-231 cells (Fig. 2F).

To investigate whether DAB2IP suppressed tumor growth in vivo, MCF7 cells stably infected with shDAB2IP lentivirus and MDA-MB-231 cells overexpressing DAB2IP were subcutaneously transplanted into female nude mice. The tumors grew faster in the MCF7-shDAB2IP group than in the control group (Supplementary Fig. 1F), whereas the overexpression of DAB2IP in MDA-MB-231 cells significantly inhibited tumor growth (Supplementary Fig. 1G). To further verify whether DAB2IP regulates glucose uptake in tumor cells in vivo, we subcutaneously transplanted MCF7 cells with control (shNC) or shDAB2IP (shDAB2IP-1#) into female BABL/c nude mice. After 4 weeks, micro-PET/CT was performed, and the mice were euthanized. The results showed that the tumor volume was larger in the MCF7-shDAB2IP-1# group than in the control group (Fig. 2G), whereas DAB2IP knockdown significantly increased 18F-FDG uptake in MCF7 xenografted tumors (Fig. 2H). These results indicate that DAB2IP decreases glucose uptake under hypoxic conditions and suppresses tumor growth in breast cancer cells.

### DAB2IP regulated glucose uptake by inducing HIF-1α ubiquitylation

**Fig. 3:**
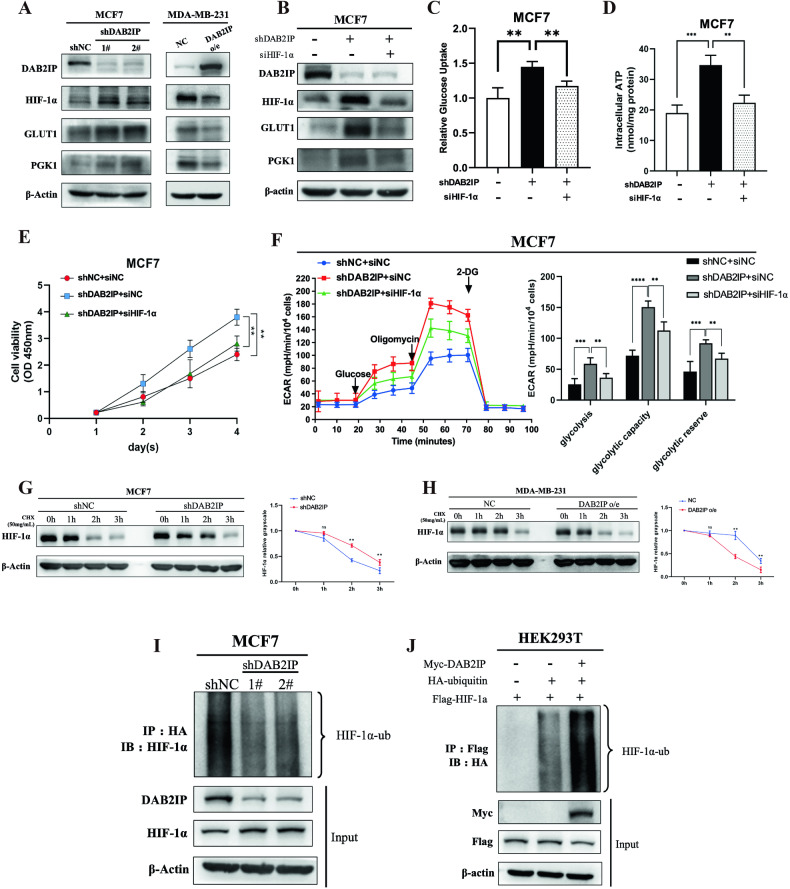
DAB2IP regulated glucose uptake by inducing HIF-1α ubiquitylation. **A** Cell lysates from MCF7 cells with DAB2IP knockdown (shDAB2IP-1#, shDAB2IP-2#) or control (shNC), and MDA-MB-231 cells with DAB2IP overexpression (DAB2IP o/e) or empty vector (NC) under hypoxic condition were analyzed via western blot assay and probed for DAB2IP, HIF-1α, GLUT1, and PGK1. β-Actin was used as loading control. **B** Cell lysates from MCF7 cells with DAB2IP knockdown (shDAB2IP) and/or HIF-1α transiently knockdown (siHIF-1α) under hypoxic condition were analyzed via western blot assay and probed for DAB2IP, HIF-1α, GLUT1, and PGK1. β-Actin was used as the loading control. **C** Glucose uptake assay was performed in MCF7 cells with DAB2IP knockdown (shDAB2IP) and/or HIF-1α (siHIF-1α) under hypoxia condition. Three bio-replications were performed for each experiment. **p < 0.01. **D** Intracellular ATP was measured in MCF7 cells with DAB2IP knockdown (shDAB2IP) and/or HIF-1α (siHIF-1α) under hypoxia condition. Three bio-replications were performed for each experiment. **p < 0.01; ***p < 0.001. **E** Cell viability was measured in MCF7 cells with DAB2IP knockdown (shDAB2IP) and/or HIF-1α (siHIF-1α) via CCK8 kit under hypoxia conditions. Three bio-replications were performed for each experiment. **p < 0.01. **F** Representative ECAR profiles of MCF7 cells with DAB2IP knockdown (shDAB2IP) and/or HIF-1α (siHIF-1α) following glucose, oligomycin and 2-DG treatments. ECAR of glycolysis, glycolytic capacity and glycolytic reverse in each group was collected and analyzed. Cells were cultured in anaerobic incubator for at least 8 h before experiment. Three bio-replications were performed for each experiment. ECAR, extracellular acidification rate; *p < 0.05; **p < 0.01; ***p < 0.001. **G** MCF7 (control or shDAB2IP) under hypoxic condition were treated with 50 μg/ml cycloheximide for the indicated times. DAB2IP and HIF-1α expression was detected via western blot assay. Hypoxia was maintained via anaerobic incubation. Quantitative analysis was conducted on HIF-1α level at different time points. Three bio-replications were performed for each experiment. ns, no significant; **p < 0.01. **H** MDA-MB-231 (control or DAB2IP overexpression) under hypoxic conditions were treated with 50 μg/ml cycloheximide for the indicated times. DAB2IP and HIF-1α expression was detected via western blot assay. Hypoxia was maintained via anaerobic incubation. Quantitative analysis was conducted on HIF-1α level at different time points. Three bio-replications were performed for each experiment. ns, no significant; **p < 0.01. **I** MCF7 (control or shDAB2IP) was transfected with HA-ubiquitin plasmid and treated with MG132 at 10 μM. Cells were cultured in anaerobic incubator for at least 8 h before experiment. HA antibody was used to detect immunoprecipitated ubiquitination proteins, and ubiquitination endogenous HIF-1α in the immunocomplexes were detected via western blot assay. **J** HEK293T was co-transfected with HA-ubiquitin, Flag-HIF-1α, and myc-DAB2IP plasmids and treated with MG132 at 10 μM for 8 h. Flag antibody was used to detect immunoprecipitated exogenous HIF-1α proteins, and ubiquitination levels of HIF-1α in the immunocomplexes were detected by HA antibody via western blot assay.

To unveil the potential pathway involved in DAB2IP-related glucose metabolism, we re-analyzed DAB2IP correlated genes in hallmark of cancer gene set (Broad institute, h1: hallmark of cancer 2019, *p* < 0.05) in TCGA-BRCA database via KEGG enrichment and found that DAB2IP was negatively associated with HIF-1α signaling (Supplementary Fig. 2A). Western blot validated that knocking down DAB2IP induced HIF-1α expression and downstream GLUT1 and PGK1 expression in MCF7, while overexpressing DAB2IP downregulated HIF-1α, GLUT1, and PGK1 in MDA-MB-231 (Fig. 3A). To confirm the role of HIF-1α in DAB2IP-regulated glucose metabolism, we first knocked down endogenous HIF-1α in MCF7 stably transfected with shDAB2IP plasmid (Fig. 3B). Our results showed that knocking down HIF-1α reversed the increase in glucose uptake (Fig. 3C), intracellular ATP production (Fig. 3D) and cell viability (Fig. 3E) triggered by shDAB2IP in MCF7 cells under hypoxic conditions. Additionally, knocking down HIF-1α significantly decreased ECAR in MCF7 transfected with shDAB2IP (Fig. 3F).

To further explore the mechanism underlying DAB2IP-suppressed HIF-1α signaling, we used cycloheximide (CHX), an inhibitor of protein synthesis, to indirectly measure the half-life of HIF-1α in breast cancer cells with different DAB2IP expression levels. The half-life of HIF-1α was significantly extended in MCF7 cells with DAB2IP knockdown (Fig. 3G) and significantly shortened in MDA-MD-231 cells overexpressing DAB2IP (Fig. 3H). Co-immunoprecipitation assay showed that either endogenous or exogenous DAB2IP bound with HIF-1α in MCF7 and HEK293T cells under hypoxic conditions (Supplementary Fig. 2B, C). Moreover, knocking down DAB2IP inhibited HIF-1α ubiquitination in MCF7 (Fig. 3I), while overexpressing DAB2IP promoted exogenous HIF-1α ubiquitination in HEK293T cells (Fig. 3J). These results indicated that DAB2IP can interact with HIF-1α and induce its ubiquitin degradation in breast cancer.

### DAB2IP interacted with E3 ubiquitin ligase STUB1

**Fig. 4:**
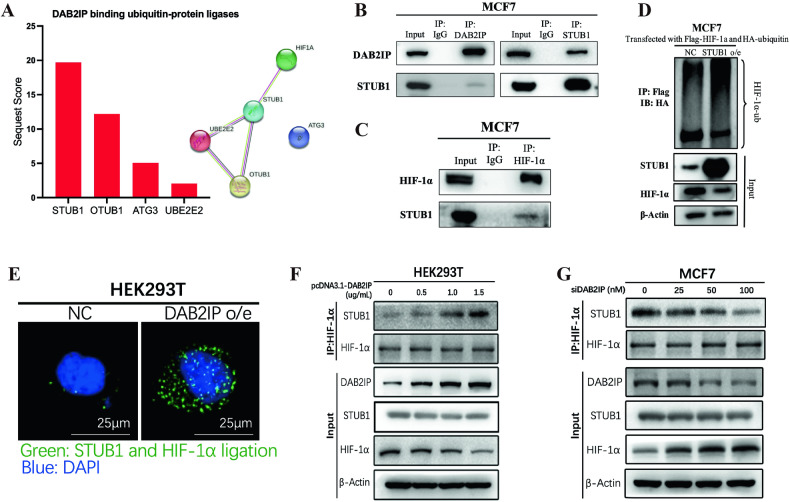
DAB2IP interacted with E3 ubiquitin ligase STUB1. **A** Sequest score of STUB1, OTUB1, ATG3, and UBE2E2 in exogeneous DAB2IP immune complex (left) from HEK293T transfected with Myc-DAB2IP. The protein–protein interaction among STUB1, OTUB1, ATG3, UBE2E2, and HIF-1α in the STRING database (right). **B** Co-immunoprecipitation with western blot assay detected STUB1 protein in DAB2IP immune complex, and DAB2IP protein in STUB1 immune complex in MCF7 cells under hypoxic condition. **C** Co-immunoprecipitation with western blot assay detected STUB1 protein in HIF-1α immune complex in MCF7 cells under hypoxic condition. **D** MCF7 (control or STUB1 overexpression) was transfected with HA-ubiquitin plasmid and Flag-HIF-1α and treated with MG132 at 10 μM. Flag antibody was used to detect immunoprecipitated HIF-1α proteins, and the ubiquitination levels of HIF-1α in the immunocomplexes were detected by HA antibody via western blot assay. **E** PLA assay was performed in HEK293T cells with control (NC) or DAB2IP overexpression (DAB2IP o/e) under hypoxic condition. Green, signals of STUB1 and HIF-1α ligation; blue, DAPI. Scale bar: 25 μm. **F** After transfecting pcDNA3.1-DAB2IP plasmids into HEK293T at the indicated concentrations under hypoxic condition, HIF-1α antibody was used to detect immunoprecipitated HIF-1α and its binding proteins. STUB1 expression in the immunocomplexes was detected by the relevant antibody via western blot assay. **G** Knocking down DAB2IP in MCF7 cells with siRNA at the indicated concentrations under hypoxic condition, HIF-1α antibody was used to detect immunoprecipitated HIF-1α and its binding proteins. STUB1 expression in the immunocomplexes was detected by relevant antibody via western blot assay.

To determine the E3 ubiquitin ligase involved in DAB2IP-induced HIF-1α ubiquitination, immunoprecipitation and mass spectrometry were performed to identify DAB2IP and HIF-1α binding proteins in HEK293T with exogeneous Myc-DAB2IP or Flag-HIF-1α overexpression. By comparing and analyzing these proteins, four E3 ubiquitin ligase (STUB1, OTUB1, ATG3, and UBE2E2) combined with both DAB2IP and HIF-1α; STUB1 represented the highest matching score in the DAB2IP immune complex in HEK293T-Myc-DAB2IP cells (Sequest score: 19.7) (Fig. 4A). Co-immunoprecipitation assays demonstrated that STUB1 interacted with both DAB2IP and HIF-1α (Fig. 4B, C). STUB1 overexpression mediated HIF-1α ubiquitination in MCF7 cells (Fig. 4D). Therefore, STUB1 was selected as a candidate E3 ubiquitin ligase for further study.

To further unveil the regulatory mechanism of DAB2IP-STUB1-HIF-1α, we re-analyzed the mass spectrum data of the STUB1 peptide sequence in the exogeneous DAB2IP and HIF-1α immune complexes from HEK293T-Myc-DAB2IP and HEK293T-Flag-HIF-1α cells, respectively. The results showed that DAB2IP bound both to the N-terminal region (56-66, 86-95, 129-140) and C-terminal region (154-167) of STUB1, while HIF-1α bound only to the C-terminal region (154-167) of STUB1 (Supplementary Fig. 2D). Considering that HIF-1α was found to interact with DAB2IP, we assumed that DAB2IP acted as a scaffold protein that coordinated the interaction between STUB1 and HIF-1α and promoted STUB1-mediated HIF-1α ubiquitination. To verify this hypothesis, Proximity ligation assay (PLA) assay was performed, revealing that DAB2IP overexpression promoted proximity ligation between STUB1 and HIF-1α (Fig. 4E). Furthermore, we transfected DAB2IP plasmid into HEK293T and found that the binding capacity of STUB1 to HIF-1α gradually increased as DAB2IP expression increased (Fig. 4F). DAB2IP knockdown reduced the interaction between STUB1 and HIF-1α in MCF7 cells (Fig. 4G).

### DAB2IP induced HIF-1α ubiquitination via STUB1

**Fig. 5:**
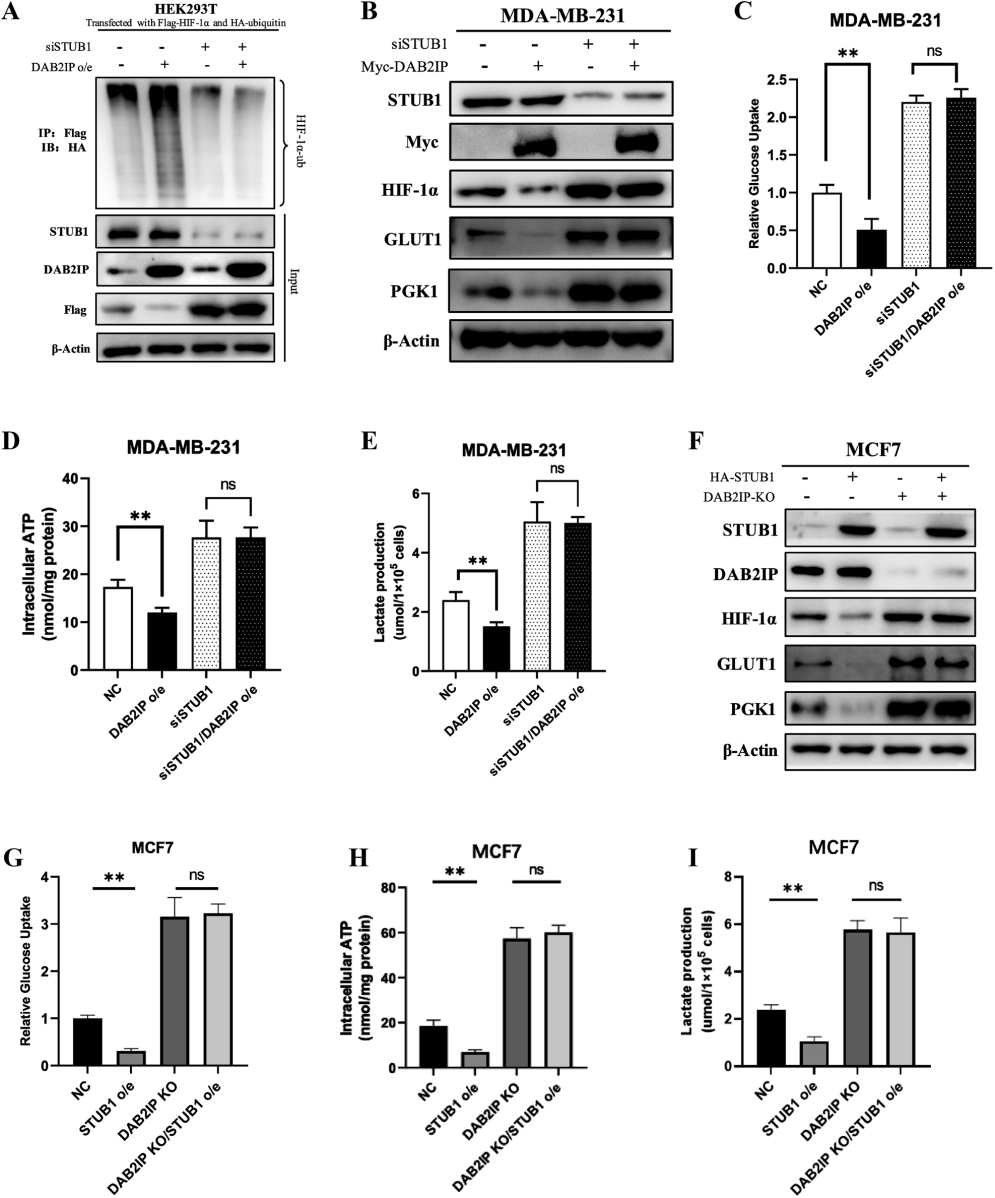
DAB2IP induced HIF-1α ubiquitination via STUB1. **A** HEK293T was co-transfected with HA-ubiquitin and Flag-HIF-1α plasmids, then overexpressed DAB2IP and/or knockdown STUB1 with relevant plasmids and siRNAs. After treating MG132 at 10 μM for 8 h, Flag antibody was used to detect immunoprecipitated exogenous HIF-1α proteins, and ubiquitination HIF-1α in the immunocomplexes were detected by HA antibody via western blot assay. **B** Cell lysates from MDA-MB-231 cells with DAB2IP overexpression (transfected with myc-DAB2IP plasmid) and/or STUB1 knockdown (siSTUB1) under hypoxic condition were analyzed via western blot assay and probed for STUB1, Myc, HIF-1α, GLUT1 and PGK1. β-Actin was used as loading control. **C** Glucose uptake assay was performed in MDA-MB-231 cells with DAB2IP overexpression (DAB2IP o/e) and/or STUB1 knockdown (siSTUB1) under hypoxia condition. Three bio-replications were performed for each experiment. **p < 0.01. **D** Intracellular ATP level was measured in MDA-MB-231 cells with DAB2IP overexpression (DAB2IP o/e) and/or STUB1 knockdown (siSTUB1) under hypoxia condition. Three bio-replications were performed for each experiment. **p < 0.01. **E** Lactate production level was measured in MDA-MB-231 cells with DAB2IP overexpression (DAB2IP o/e) and/or STUB1 knockdown (siSTUB1) under hypoxia condition. Three bio-replications were performed for each experiment. **p < 0.01. **F** Cell lysates from MCF7 cells with DAB2IP knockout (DAB2IP-KO) and/or STUB1 overexpression (transiently transfected with HA-STUB1) under hypoxic condition were analyzed via western blot assay and probed for STUB1, DAB2IP, HIF-1α, GLUT1 and PGK1. β-Actin was used as loading control. **G** Glucose uptake assay was performed in MCF7 cells with DAB2IP knockout (DAB2IP-KO) and/or STUB1 overexpression (transfected with HA-STUB1) under hypoxic condition. Three bio-replications were performed for each experiment. **p < 0.01. **H** Intracellular ATP level was measured in MCF7 cells with DAB2IP knockout (DAB2IP-KO) and/or STUB1 overexpression (transfected with HA-STUB1) under hypoxic condition. Three bio-replications were performed for each experiment. **p < 0.01. **I** Lactate production level was measured in MCF7 cells with DAB2IP knockout (DAB2IP-KO) and/or STUB1 overexpression (transfected with HA-STUB1) under hypoxic condition. Three bio-replications were performed for each experiment. **p < 0.01.

Next, we validated whether DAB2IP induced HIF-1α ubiquitination through STUB1. The ubiquitination assay revealed that knocking down endogenous STUB1 significantly reversed DAB2IP induced HIF-1α ubiquitination in HEK293T cells (Fig. 5A). Similarly, knocking down endogenous STUB1 in MDA-MB-231 cells reversed DAB2IP induced HIF-1α and downstream GLUT1 and PGK1 expression (Fig. 5B). Phenotypic experiments showed that STUB1 knockdown inhibited glucose uptake and intracellular ATP and lactate production in DAB2P-overexpressing MDA-MB-231 cells (Fig. 5C–E). Conversely, STUB1 overexpression induced HIF-1α, GLUT1, and PGK1 expression (Fig. 5F) and upregulate glucose uptake rate and intracellular ATP/lactate production in DAB2IP-knockout MCF7 cells (Fig. 5G–I). We also validated the role of HIF-1α in DAB2IP-regulated glucose metabolism. Western blot assay and phenotype experiments showed that overexpressing HIF-1α significantly induced GLUT1 and PGK1 expression (Supplementary Fig. 2E) and upregulated glucose uptake rate (Supplementary Fig. 2F) and intracellular ATP/lactate production (Supplementary Fig. 2G, H) in DAB2IP overexpressing MDA-MB-231 cells. Thus, we concluded that DAB2IP may regulate HIF-1α related glucose metabolism in a STUB1-mediated ubiquitination dependent manner.

### PER domain of DAB2IP was essential for STUB1-mediated HIF-1α ubiquitination

**Fig. 6:**
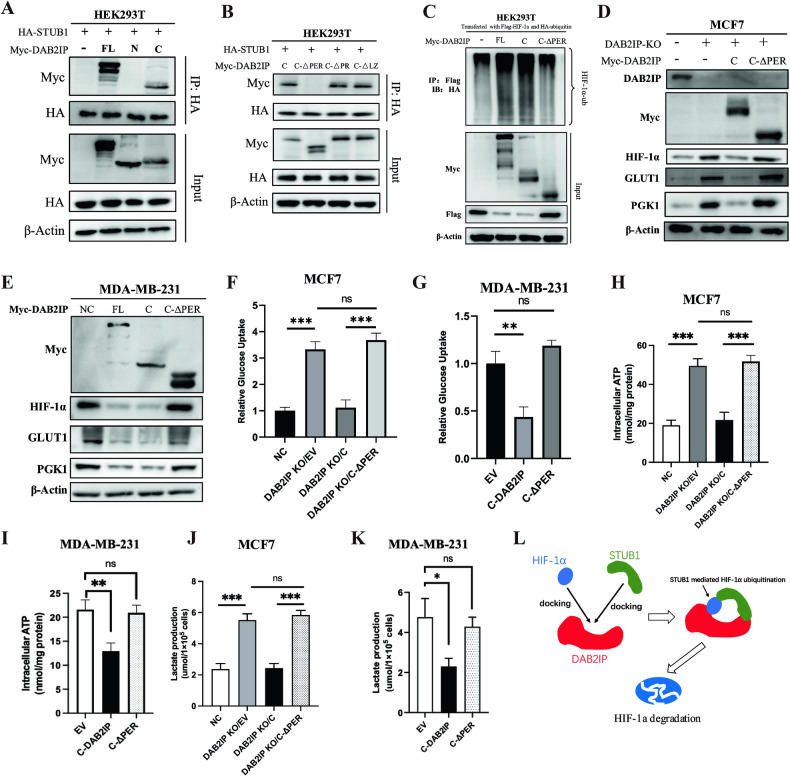
PER domain of DAB2IP was essential for STUB1-mediated HIF-1α ubiquitination. **A** HEK293T was co-transfected with HA-STUB1 and different myc-DAB2IP mutants (FL, N or C). Myc antibody was used to immunoprecipitated exogenous DAB2IP proteins, and STUB1 in the immunocomplexes were detected by HA antibody via western blot assay. FL, full length; N, N-terminal; C, C-terminal. Details of mutants are shown in Supplementary Fig. 2I. **B** HEK293T was co-transfected with HA-STUB1 and different myc-DAB2IP mutants (C, C-△PER, C-△PR or C-△LZ). Myc antibody was used to immunoprecipitated exogenous DAB2IP proteins, and STUB1 in the immunocomplexes were detected by HA antibody via western blot assay. Details of mutants are shown in Supplementary Fig. 2J. **C** HEK293T was co-transfected with HA-ubiquitin and Flag-HIF-1α plasmids, then overexpressed DAB2IP and its mutants (FL, C, C-△PER) with relevant plasmids. After treated with MG132 at 10 μM for 8 h, Flag antibody was used to immunoprecipitated exogenous HIF-1α proteins, and ubiquitination HIF-1α in the immunocomplexes were detected by HA antibody via western blot assay. **D** Cell lysates from MCF7 cells with DAB2IP knockout (via CRISPR/Cas9) and/or DAB2IP mutants’ overexpression (transfected with myc-DAB2IP-C and myc-DAB2IP-C-△PER plasmids) under hypoxic conditions were analyzed via western blot assay and probed for DAB2IP, Myc, HIF-1α, GLUT1 and PGK1. β-Actin was used as loading control. **E** Cell lysates from MDA-MB-231 cells with DAB2IP wild-type (transfected with myc-DAB2IP-FL) and mutants’ overexpression (transfected with myc-DAB2IP-C, myc-DAB2IP-C-△PER plasmids) under hypoxic conditions were analyzed via western blot assay and probed for Myc, HIF-1α, GLUT1, and PGK1. β-Actin was used as loading control. **F** Glucose uptake assay was performed in MCF7 cells with endogenous DAB2IP knockout (DAB2IP KO), empty vector (EV), myc-DAB2IP-C-terminal mutant (C) and C-△PER mutant overexpression under hypoxia conditions. Three bio-replications were performed for each experiment. ***p < 0.001. **G** Glucose uptake assay was performed in MDA-MB-231 cells with EV, myc-DAB2IP-C-terminal mutant (C-DAB2IP) and C-△PER mutant overexpression under hypoxia conditions. Three bio-replications were performed for each experiment. **p < 0.01. **H** Intracellular ATP level was measured in MCF7 cells with endogenous DAB2IP knockout (DAB2IP KO), EV, myc-DAB2IP-C-terminal mutant (C), and C-△PER mutant overexpression under hypoxia condition. Three bio-replications were performed for each experiment. ***p < 0.001. **I** Intracellular ATP level was measured in MDA-MB-231 cells with EV, myc-DAB2IP-C-terminal mutant (C-DAB2IP) and C-△PER mutant overexpression under hypoxia conditions. Three bio-replications were performed for each experiment. **p < 0.01. **J** Lactate production level was measured in MCF7 cells with endogenous DAB2IP knockout (DAB2IP KO), EV, myc-DAB2IP-C-terminal mutant (C), and C-△PER mutant overexpression under hypoxia condition. Three bio-replications were performed for each experiment. ***p < 0.001. **K** Lactate production level was measured in MDA-MB-231 cells with EV, myc-DAB2IP-C-terminal mutant (C-DAB2IP) and C-△PER mutant overexpression under hypoxia condition. Three bio-replications were performed for each experiment. *p < 0.05. **L** Model for the DAB2IP inducing HIF-1a ubiquitylation degradation in breast cancer.

To determine the key domain of DAB2IP which involved in STUB1-mediated HIF-1α ubiquitination, three previously established DAB2IP truncated mutants (full length: 1-1189; N-terminal: 1-563; C-terminal: 564-1189) [16] were used to screen the potential DAB2IP region that interacted with STUB1 (Supplementary Fig. 2I). Co-immunoprecipitation assays showed that the DAB2IP C-terminal mutant pulled down STUB1 in HEK293T cells, whereas the N-terminal mutant did not (Fig. 6A). The C-terminal segment of DAB2IP contains three domains: a period-like domain (PER: 591-719), a proline-rich domain (PR: 727-736), and a leucine zipper domain (LZ: 842-861) [13]. Therefore, we established PER (△PER), PR(△PR), and LZ (△LZ) domain deletion based on DAB2IP C-terminal truncated mutation plasmid (Supplementary Fig. 2J). When PER was deleted, the C-terminal segment of DAB2IP neither immunoprecipitated STUB1 nor induced HIF-1α ubiquitination in HEK293T cells (Fig. 6B, C). Moreover, compared with the full length and C-terminal segments of DAB2IP, the PER depletion mutant did not inhibit HIF-1α and its downstream expression in MCF7 with DAB2IP knockout (DAB2IP-KO) cells and MDA-MB-231 cells (Fig. 6D, E). Glucose uptake and intracellular ATP/lactate production assays also showed that the PER domain was essential for DAB2IP inhibited glucose uptake (Fig. 6F, G), intracellular ATP production (Fig. 6H, I), and lactate production (Fig. 6J, K) in both MCF7 and MDA-MB-231 cells. In summary, our study revealed a potential mechanism by which DAB2IP can act as a scaffold to promote the interaction between HIF-1α and STUB1, thus inducing HIF-1α ubiquitylation degradation and inhibiting glucose uptake in breast cancer cells under hypoxic conditions (Fig. 6L).

## Discussion

DAB2IP is an important tumor suppressor in multiple key oncogenic pathways, including TNFα/NF-κB, WNT/β-catenin, PI3K/AKT, and androgen receptors; therefore, inactivated DAB2IP is widely reported to trigger tumor initiating and cancer progressing [13, 15]. As a scaffold protein, DAB2IP can function as a competitor or scavenger by binding to multiple signaling regulators and preventing their interaction with other up/downstream effectors, thus potentially modulating a remarkable array of cancer-related pathways [13]. Our previous studies unveiled the tumor suppression effect and mechanism of DAB2IP in prostate cancer [20–22] and demonstrated that DAB2IP inhibited cell proliferation, epithelial–mesenchymal transition (EMT), and stem cell–like features in human colorectal cancer, especially in the p53 wild-type subgroup [16, 23]. Additionally, we reported that DAB2IP inhibits invasiveness and metastasis in breast cancer by inhibiting invadopodia formation [17]. Currently, reprogrammed glucose metabolism appears to be a hallmark of cancer [24] and can often increase the hypoxic adaptation of tumor cells, thus leading to poor patient prognosis [25]. Some studies have reported that DAB2IP may be involved in hypoxia-related regulation in breast and liver cancer [26, 27]. However, the precise role of DAB2IP in the metabolic reprogramming of tumors under hypoxic conditions remains unclear.

In this study, we revealed that DAB2IP decreases glucose uptake under hypoxic conditions, thereby inhibiting glycolysis and intracellular ATP production in breast cancer. Considering the critical role of glucose in the energy supplementation of cancer cells, intervention with DAB2IP is a potential strategy for mediating tumor-related metabolic reprogramming, thus limiting cancer progression over a wide spectrum. Our study also demonstrated that DAB2IP may downregulate glucose transporter 1 (GLUT1) and its downstream PGK1 by suppressing HIF-1α signaling. Coincidently, Zhou et al. reported that loss of DAB2IP induced HIF-2α expression by activating the mTOR pathway in renal cell cancer [28], while Wang et al. found that DAB2IP regulated EMT by destabilizing HIF-1α in prostate cancer [29]. However, little data has been reported on the detailed biological function of DAB2IP in HIF-1α-related signaling. The present study provides evidence that DAB2IP may induce HIF-1α degradation in an ubiquitylation-dependent manner. Moreover, we have identified the ubiquitin E3 ligase STUB1, which was found to be essential in the mechanism by which DAB2IP mediates HIF-1α ubiquitylation. Our results reveal a new possibility that DAB2IP mediates tumor suppression in breast cancer.

Ubiquitylation is a crucial component of post-translational modifications that affects the function of target proteins during both healthy and pathological events [30]. In HIF-1α signaling, ubiquitylation plays an essential role in determining the half-life and transcriptional activity of HIF-1α, thus controlling the hypoxic response of mammalian cells [31]. Multiple oncogenic pathways, including CDC20/PHD3 [32], SENP1/USP28 [33], and ATF4/pVHL [34] have been demonstrated to dysregulate HIF-1α ubiquitylation and induce tumor progression. In our current research, we found that DAB2IP induced HIF-1α ubiquitylation and degradation through STUB1. STUB1 (also known as CHIP) is an essential E3 ligase involved in protein quality control that degrades oncoproteins to exert tumor-suppressive functions [35]. Recently, STUB1 has emerged as an important regulator of aging, autophagy, and cancer immunity [35]. In 2010, Luo et al. and Bento et al. observed that STUB1 selectively mediated the ubiquitylated degradation of HIF-1α [36, 37]; however, the mechanism by which this occurs requires further investigation. Our finding showed that DAB2IP may act as a platform to recruit STUB1 and HIF-1α and can induce STUB1-mediated HIF-1α degradation by promoting their interaction. These results partially revealed the regulatory mechanism of STUB1-mediated ubiquitylation. Whether DAB2IP is involved in STUB1-driven ubiquitination of other oncogenic proteins should be explored in the future.

Another question raised in this study is the paradoxical role of DAB2IP in regulating protein ubiquitylation. In this study, DAB2IP functioned as an inducer of STUB1-driven HIF-1α ubiquitylation. However, our previous study showed that DAB2IP inhibited wild-type p53 ubiquitination by antagonizing GRP75 in colorectal cancer [16]. DAB2IP induces ALK deubiquitination through USP10 in breast cancer [17]. Similarly, other studies have reported that DAB2IP induces PARP-1 protein ubiquitylation [38], whereas it inhibits p27 protein ubiquitylation [39] in renal carcinoma. The biological function of DAB2IP in regulating ubiquitination and protein stabilization must be evaluated in the future.

As a scaffold protein, DAB2IP consists of several domains, including the pleckstrin homology (PH) domain, PKC-conserved region 2 (C2) domain, Ras-GTPase-activating protein (Ras-GAP) domain, C-terminal period-like (PER) domain, proline-rich (PR) domain, and the leucine zipper motif (LZ) [15]. In this research, we identified the PER domain of DAB2IP was essential for STUB1 mediated HIF-1α ubiquitination. The PER domain is a non-described region involved in protein–protein interactions [13]. However, the biological role of the PER domain of DAB2IP remains unclear. Zhang et al. reported that it interacted with TRAF2, thus inhibiting NF-kB activity [40], while Zhang et al. observed that the PER of DAB2IP displaced the binding between 14-3-3 and ASK1 protein, thereby enhancing ASK1 auto-phosphorylation and pro-apoptotic signals [41]. Furthermore, the PER domain may also be important for modulating PI3K-Akt activity, thereby connecting both survival and death signals and maintaining homeostasis in prostate cancer cells [20]. This study showed that DAB2IP interacted with STUB1 and induced HIF-1α ubiquitylated degradation via the PER domain. Interestingly, we previously found that DAB2IP binds to GRP75 and enhances wild-type p53 stability via its Ras-GAP domain [16]. The specific functions of the different domains in DAB2IP-mediated ubiquitination regulation require further investigation.

This study had some limitations. We found that loss of DAB2IP significantly induced glucose uptake, subsequently increasing lactate and intracellular ATP production in breast cancer cells under hypoxic conditions. However, the influence of DAB2IP expression on glucose metabolism under normoxic conditions remains unclear. The reason may be that DAB2IP regulates glucose metabolism via HIF-1α signaling, which is significantly induced by hypoxia. Future studies should validate other potential pathways involved in metabolic reprogramming. Second, although we found that DAB2IP inhibited GLUT1 and PGK1 expression, thereby affecting ECAR in breast cancer cells, other key enzymes involved in glucose metabolism, such as hexokinase, glucose-6-phosphate isomerase, phosphofructokinase, pyruvate dehydrogenase, and lactate dehydrogenase A/B (LDHA/B) [42], were not well investigated. Third, tumor cells derive the energy required for uncontrolled replication by rapidly consuming glucose and converting it to lactate (Warburg effect) [43]. Although the loss of DAB2IP induces lactate and ATP production, it remains uncertain whether DAB2IP subsequently regulates glycolysis or the Warburg effect in breast cancer cells. Finally, the PER domain has been identified as a key region and potential target for DAB2IP-mediated glucose metabolism. Therefore, additional studies are required to synthesize short peptides or small molecular compounds targeting this motif and to prove its therapeutic value.

In conclusion, our research revealed a novel function of DAB2IP on STUB1-mediated HIF-1α ubiquitylated degradation and glucose metabolic reprogramming in breast cancer. These findings enrich our understanding of the crosstalk between DAB2IP and the hypoxia signaling pathway and provide new potential targets for cancer therapy.

## Materials/subjects and methods

### Clinical breast cancer specimens and IHC

Between December 2018 and December 2021, 24 breast cancer patients from Tongji Hospital, Huazhong University of Science and Technology underwent 18F-FDG PET/CT scans, and tumor biopsies were retrospectively collected and analyzed. The maximum standard uptake value (SUVmax) of the primary tumor was obtained from a PET/CT scan and analyzed at the Department of Radiology, Tongji Hospital. Paraffin sections of primary tumors were also obtained from these patients through surgical resection or biopsy, and IHC staining was performed using the DAB2IP antibody (Abcam, Cat. #ab87811), as previously described [16].

The IHC results were analyzed under a microscope at 200x magnification. For each specimen, five visual fields were randomly selected and scored by summing the proportions and staining scores. The proportion score reflected the fraction of positively stained cells (0, none; 1, ≤ 25%; 2, 25% to 50%; 3, 50% to 75%; 4, > 75%), and the staining score revealed staining intensity (0, no staining; 1, weak; 2, intermediate; 3, strong). DAB2IP IHC intensity was calculated as the sum of the proportion and staining scores. The final DAB2IP IHC intensity score was the average score of five visual fields.

### Cell lines, culture, and hypoxia induction

Breast cancer cell lines MCF7, T47D, ZR-75-1, MDA-MD-231, MDA-MD-468, HCC1937, and human embryonic kidney cell line HEK293T were purchased from and authenticated by the American Type Culture Collection (Manassas, VA, US). Cells were cultured at 37 °C in a 5% CO_2_ incubator. The MCF7 and HEK293T cells were cultured in Dulbecco’s modified Eagle’s medium (DMEM) (KeyGEN, Cat. #KGM12800N). T47D, ZR-75-1, and HCC1937 cells were cultured in RPMI-1640 medium (1640) (KeyGEN, Cat. #KGM31800N). The MDA-MB-231 and MDA-MB-468 cells were cultured in Leibovitz L-15 medium (L15) (KeyGEN, Cat. #KGM41300N). All culture media were supplemented with 10% fetal bovine serum (Cat. #086-150) and 1% penicillin/streptomycin (Cat. #KGY0023). Mycoplasma contamination was test by Mycoplasma PCR Detection Kit (Beyotime, Cat. #C0301S) according to the manufacturer’s instruction. To stimulate hypoxic condition, cells were cultured in anaerobic incubator (5% CO_2,_ 1% O_2_) at 37 °C for 12 h before further experiments were conducted.

### Small interfering RNA, plasmid, and site-directed mutagenesis

Small interfering RNAs (siRNAs) were purchased from Tsingke Biotechnology Co., Ltd. (Beijing, China). pcDNA3.1-Flag-tagged-DAB2IP, pcDNA3.1-Myc-tagged-DAB2IP, pcDNA3.1-Myc-tagged-DAB2IP-C-terminal, and pcDNA3.1-Myc-tagged-DAB2IP-N-terminal overexpressing plasmids were obtained as previously described [16]. HIF-1α and STUB1 overexpressing plasmids were purchased from TsingkeBiotechnolog Co., Ltd. (Beijing, China). Truncated and site-directed mutagenesis of DAB2IP plasmids was performed using the Mut Express II Fast Mutagenesis Kit V2 (Vazyme, Cat. # C214-01) according to the manufacturer’s protocol. The siRNAs sequences and primers used for plasmid mutagenesis are listed in S. Table 1.

### CRISPR/Cas9

DAB2IP was knocked out using the CRISPR/Cas technique. Briefly, sgRNAs targeting DAB2IP were designed using the website software E-CRISP (http://www.e-crisp.org/E-CRISP/) (target sequence: 5‘–3‘ GGAGGTCCTCCTACTACTAC). Then, the sgRNA was synthesized and packaged into a CRISPR/Cas9 lentivirus (performed by Shanghai Genechem Co., Ltd). After infection, the cells were treated with 2 μg/mL puromycin for 1 week. The remaining cells were trypsinized and seeded into 96-well plates to obtain single-cell clones. After single-cell expansion, western blotting was performed to identify the DAB2IP-KO clones.

### Transfection and stable cell line establishment

Transient transfection of small interfering RNA (siRNA) and plasmids were performed using Lipofectamine 3000 (Thermo Fisher, Cat. #L3000015) according to the manufacturer’s protocol. Biological and biochemical experiments were performed 48 h after transfection.

Recombinant lentiviruses expressing sh-scramble and sh-DAB2IP (previously obtained in [16]) were used to establish stable DAB2IP knockdown (DAB2IP-KD) cell lines. Stable DAB2IP overexpressing lentivirus was established using pLVX (backbone) - psPAX2/pMD2. G (packaging vector). The DAB2IP gene from the pcDNA3.1-DAB2IP plasmid was cloned into the pLVX vector, and the virus particles were packaged using HEK293T. At 48 h after lentivirus infection, cells were treated with 2 μg/mL puromycin until all cells in blank group had died. Western blotting was used to validate the DAB2IP protein levels in stable cell lines.

### Cell viability examination

Briefly, 5000 cells per well were seeded in 96-well plate and treated with CoCl_2_ at a concentration of 150 μM to induce hypoxia. Then the cells were cultured at 37 °C in a 5% CO_2_ incubator for 3 days. The cell viability was examined at 24, 48, and 72 h. During each examination, medium was replaced and added 10 μL Cell Counting Kit-8 (CCK8) solution (MCE, Cat. #: HY-K0301). After 120 min of incubation at 37 °C, 450 nm optical density (OD) was detected via spectrometer.

### Glucose uptake assay

The glucose uptake assay was performed using a Glucose Uptake Assay Kit (Promega, Cat. #: J1341). Briefly, 2-Deoxy-D-glucose (2-DG) was added to the culture medium. After 10 min incubation at 37 °C, the 25 μL reaction stop solution, 25 μL neutralization buffer, and 100 μL 2-Deoxy-D-glucose 6-phosphate (2-DG-6P) solution provided by the kit were sequentially added into the medium according to the manufacturer’s instruction. Then, the luminescence signal was detected and recorded using a GloMax® 20/20 Luminometer. Three bio-replications were performed for each experiment.

### Intracellular adenosine triphosphate (ATP) examination

The intracellular ATP levels were measured using an ATP detection kit (Beyotime, Cat. #S0026). Briefly, 2 × 10^6^ cells were collected and lysed in 200 μL ATP detection lysis buffer 4 °C for 10 min. Then cell lysate was centrifuged at 4 °C, 12000 g for 5 min, and the supernatant was collected. Working solutions and standards were prepared according to the manufacturer’s instructions. The detection working solution was mixed with the supernatants or standards at 4 °C for 5 min, and the luminescence intensity was measured by GloMax® 20/20 Luminometer. Three bio-replications were performed for each experiment.

### Lactate production examination

The culture medium was collected and centrifuged at 14,000 g at 4 °C for 5 min. Then, the lactate level in each supernatant was measured with a lactate content detection kit (Nanjing Jian-Cheng Biotech Co., Ltd, Cat. #A019-2-1) using a multimode plate reader according to the manufacturer’s instructions.

### Seahorse ECAR examination

A Seahorse XF Pro analyzer (Agilent, US) was used to detect ECAR. Briefly, 5 × 10^5^ cells per well were seeded into an XF24 plate, and the XF flux sensor was hydrated without CO_2_ overnight according to the manufacturer’s protocol. On the day of the experiment, the sensor cartridge of the instrument was pre-warmed at 37 °C for 5 h. The sample culture medium was replaced by with analytical buffer, and the XF24 plate was incubated without CO_2_ at 37 °C for 60 min. The XF24 plate was then placed into the instrument, and the measurement was started. Glucose, oligomycin, and 2-DG were obtained from a Glycolysis Stress Test Kit (Agilent, Cat. #:103020) were sequentially added to the sample at appropriate concentrations, according to the manufacturer’s instructions. ECAR values were recorded and analyzed using the GraphPad Prism software. Each experiment was performed in triplicate.

### Western blot and co-immunoprecipitation (Co-IP) assay

Western blotting and co-IP assays were performed as previously described [16]. The antibodies used in the experiments are listed in S. Table 2.

### Ubiquitination assay

The ubiquitination assay was performed as previously described [16]. Briefly, cells transfected with HA-tagged-ubiquitin plasmid were treated with MG-132 at a dose of 10 μM for 8 h. Then cells were lysed in NP40 buffer and centrifuged at 12000 g at 4 °C for 15 min. Cell lysates were immunoprecipitated with the agarose beads which were incubated with indicated primary antibody overnight at 4 °C. Target protein ubiquitination was examined by western blot using an anti-HA antibody.

### PLA

Cells were fixed with 4% paraformaldehyde at 25 °C for 20 min and were permeabilized with 0.1% Triton-X100 for 10 min. Nonspecific staining was blocked by incubation in goat serum for 1 h. Subsequently, cells were incubated with the primary antibody STUB1 (C3B6, CST Cat# 2080, RRID: AB_2198052) at 1:100 dilution at 4 °C overnight. After washing, hybridization, ligation, amplification and staining were performed by PLA detection kit (Duolink® PLA Control Kit – PPI, Sigma Aldrich, Cat. # DUO92202) according to the manufacturer’s protocol. Images were captured using a fluorescence microscope (OLYMPUS IX71) at 400 magnification. The PLA was visualized using a green signal.

### Mass spectrum analysis

Mass spectral analysis was performed by Shanghai Lu-Ming Biotech Co., Ltd., as previously described [16]. Mass spectra were analyzed using Proteome Discoverer 2.3 search engine (Thermo Scientific, MA, US), and Uniprot-Homo database using the Sequest algorithm [44].

### Animal study

All animal experiments were approved by the Ethics Committee of Tongji Hospital. Female BABL/c nude mice (age 4 weeks) were purchased from Shulaibao (Wuhan) Biotechnology Co., Ltd. The mice were randomly divided into two groups without blinding and subcutaneously injected 1 × 10^6^ tumor cells in Matrigel (Corning), with six mice per group. After 30–36 days, the mice were euthanized, and the tumors were removed, embedded, sliced, and stained with hematoxylin and eosin (HE). The expression of DAB2IP was evaluated using immunohistochemistry.

A micro-PET/CT scan was performed by skilled technicians in the Nuclear Medicine Department of Wuhan Union Hospital. Briefly, the female BABL/c nude mice were randomly divided into two groups without blinding (*n* = 3 per group) and subcutaneously injected 1 × 10^6^ tumor cells in Matrigel (Corning). After 4 weeks, the mice were fasted for 8 h before scanning. After weighting, 100 μL of 1.85 MBq 18F-FDG/PBS solution was injected into the mice via caudal vein at 1 h before experiment. Mice were anesthetized with 5% isoflurane and placed on a scanning bed. During scanning, an airflow of 1% isoflurane was used to maintain anesthesia. The CT and PET scan images were reconstructed using uBio-EXPLORER software through the Order Subset Expectation Maximization (OSEM) algorithm (four OSEM iterations with 1.4 mmHD resolution) and CT attenuation correction. The SUVmax of the xenografted tumors was also measured and analyzed.

### Bioinformatics and statistical analysis

Gene expression profile and prognosis data of patients with breast cancer was obtained from The Cancer Genome Atlas – breast invasive carcinoma (TCGA-BRCA) database (http://portal.gdc.cancer.gov) and analyzed via GEPIA software (http://gepia.cancer-pku.cn/index.html) [45] and R2: Genomics Analysis and Visualization Platform (http://hgserver1.amc.nl/cgi-bin/r2/main.cgi). Potential protein interactions were analyzed using the STRING database (https://www.string-db.org/).

All continuous data were reported as the mean ± SD and analyzed via Two-tailed Student’s *t*-test or analysis of variance (ANOVA). Categorical variables were compared using the χ2 test or Fisher test. *P* < 0.05 was considered statistically significant. Statistical analysis was performed using the software package SPSS (version 19.0 for Windows; IBM, USA) and GraphPad Prism (version 9.41 for Mac; GraphPad Software, Inc., USA).

### Supplementary information

SUPPLEMENTAL MATERIAL

## Data Availability

The datasets used and/or analyzed in the present study are available from the corresponding author upon reasonable request.
