# Supplementary material for: DAB2IP inhibits glucose uptake by modulating HIF-1α ubiquitination under hypoxia in breast cancer
Source: Oncogenesis. 2024 Jun 11;13(1):20. doi: 10.1038/s41389-024-00523-4 (PMC11166643; doi:10.1038/s41389-024-00523-4)
Supplement: Supplementary file 1 — SUPPLEMENTAL MATERIAL [file 41389_2024_523_MOESM1_ESM.docx]

**S.Fig.1. DAB2IP regulated glucose metabolism and tumor growth in breast cancer.**


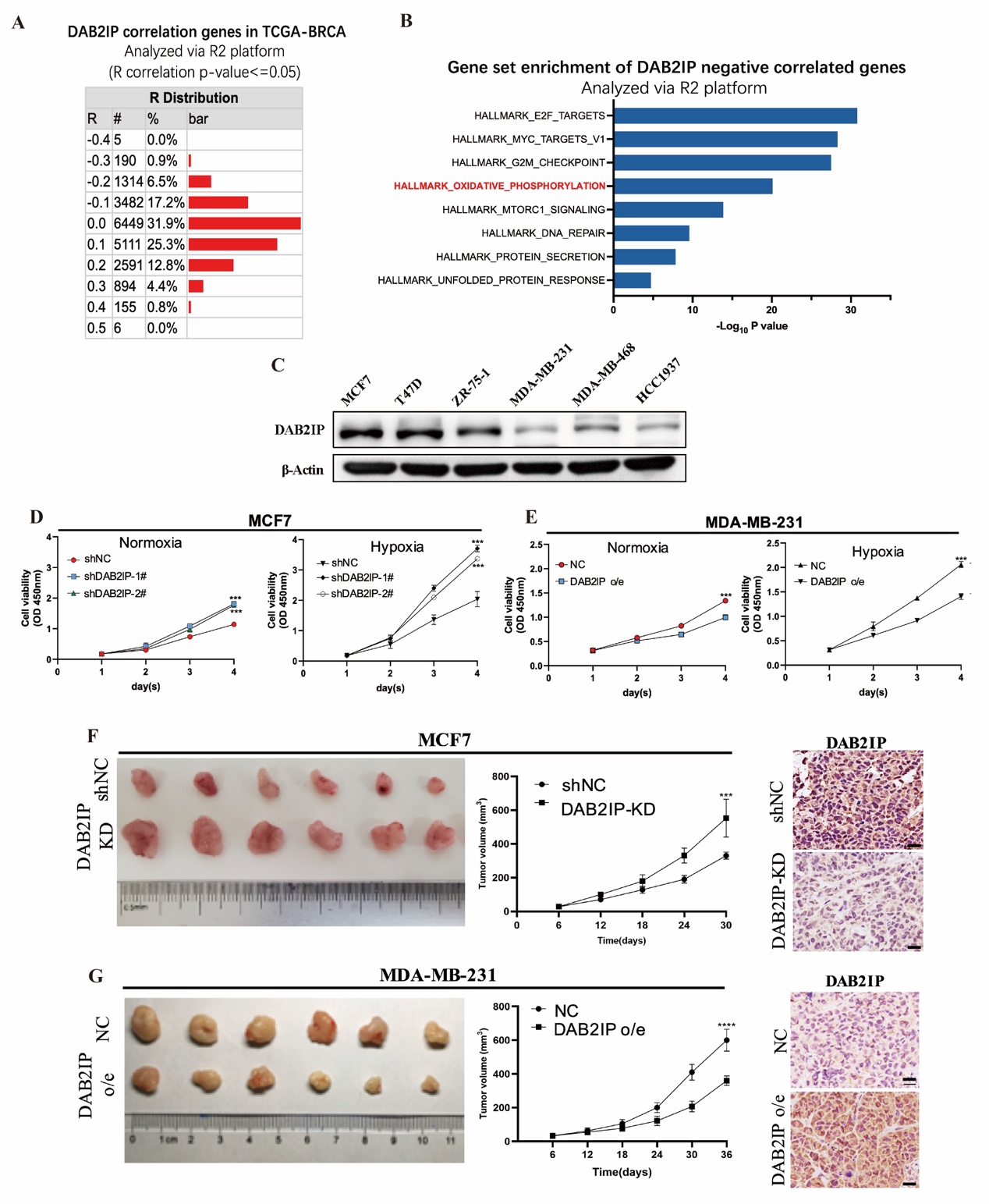


(A) Distribution of DAB2IP correlated genes in TCGA-BRCA dataset. Pearson correlation analysis of gene expression between DAB2IP and other probes in TCGA-BRCA database were performed via R2: Genomics Analysis and Visualization Platform. Correlation genes with p-value≤0.05 were selected and shown in this panel. # gene counts; % gene proportion.

(B) Gene set enrichment of DAB2IP negative correlated genes from TCGA-BRCA (R<0, p<0.05). Analysis was performed via R2: Genomics Analysis and Visualization Platform using “h1: hallmark of cancer” Molecular Signatures Database (MSigDB).

(C) Western blot assay verifying DAB2IP expression in six breast cancer cell lines (MCF7, T47D, ZR-75-1, MDA-MB-231, MDA-MB-468, HCC1937). β-Actin was used as loading control.

(D) Cell viability was measured in MCF7 cells with DAB2IP knockdown (shDAB2IP-1#, shDAB2IP-2#) via CCK8 kit under normoxic and hypoxia condition. Hypoxia was triggered via anaerobic incubation for at least 8 h. Three bio-replications were performed for each experiment. ***p<0.001.

(E) Cell viability was measured in MDA-MB-231 cells with DAB2IP overexpression (DAB2IP o/e) via CCK8 kit under normoxic and hypoxia condition. Hypoxia was triggered via anaerobic incubation for at least 8 h. Three bio-replications were performed for each experiment. ***p<0.001.

(F) Four-week-old female BABL/c nude mice were subcutaneously injected 1x10^6^ MCF7 with DAB2IP knock down cells (DAB2IP-KD) or control (shNC) (n=6 per group). Tumor volume was measured per 6 days after injection. After 30 days, the mice were euthanized, and the tumors were removed. DAB2IP expression level in xenografted tumor was verified via immunohistochemistry staining. Scale bar: 20 μm; ***p<0.001.

(G) Four-week-old female BABL/c nude mice were subcutaneously injected 1x10^6^ MDA-MB-231 cell with DAB2IP overexpression (DAB2IP o/e) or control (NC) (n=6 per group). Tumor volume was measured per 6 days after injection. After 36 days, the mice were euthanized, and the tumors were removed. DAB2IP expression level in xenografted tumor was verified via immunohistochemistry staining. Scale bar: 20 μm; ***p<0.001.

**S.Fig.2. DAB2IP regulated glucose uptake through HIF-1α signaling.**

**
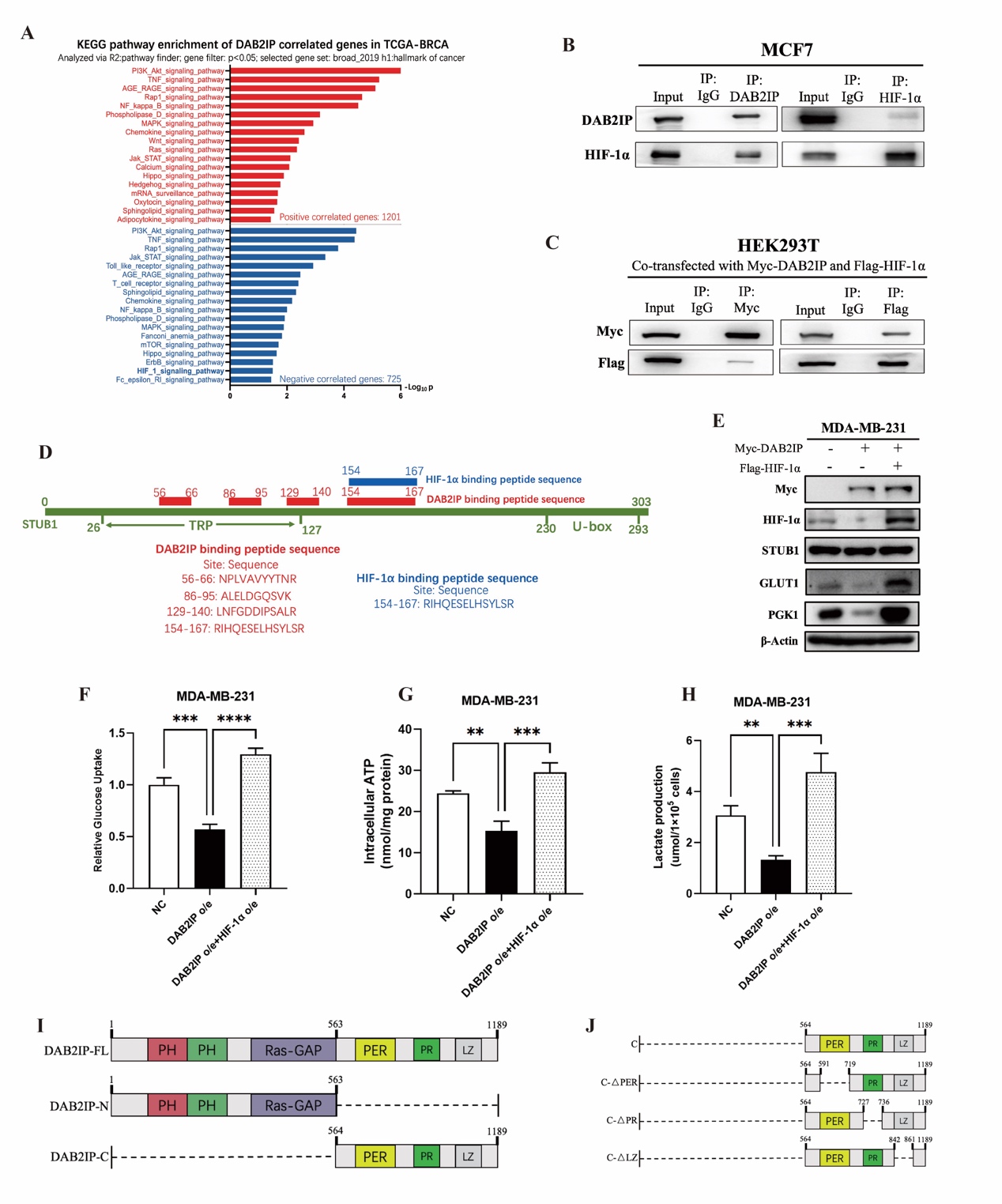
**

(A) KEGG pathway enrichment analysis of DAB2IP correlated genes in TCGA-BRCA. Genes in “h1: hallmark of cancer” gene-set with Pearson correlation p value<0.05 were selected and analyzed using R2: pathway finder.

(B) Coimmunoprecipitation with Western blot assay detected HIF-1α protein in DAB2IP immune complex, and DAB2IP protein in HIF-1α immune complex in MCF7 cells under hypoxic condition.

(C) HEK293T was co-transfected with Myc-DAB2IP and Flag-HIF-1α plasmids, then coimmunoprecipitation with Western blot assay detected exogenous HIF-1α protein (Flag) in exogenous DAB2IP (Myc) immune complex, and exogenous DAB2IP protein (Myc) in exogenous HIF-1α (Flag) immune complex in HEK293T cells.

(D) Diagram of DAB2IP binding site and HIF-1α binding site on STUB1 protein. Data were obtained from the mass spectrum analysis of STUB1 peptide sequence in DAB2IP immune complex (red) in HEK293T-Myc-DAB2IP cells and HIF-1α immune complex (blue) in HEK293T-Flag-HIF-1α cells.

(E) Cell lysates from MDA-MB-231 cells with DAB2IP overexpression (Myc-DAB2IP) and/or HIF-1α overexpression (Flag-HIF-1α) under hypoxic condition were analyzed using Western blot and probed for Myc, HIF-1α, STUB1, GLUT1 and PGK1. β-Actin was used as loading control.

(F) Glucose uptake assay was performed in MDA-MB-231 cells with DAB2IP overexpression (Myc-DAB2IP) and/or HIF-1α overexpression (Flag-HIF-1α) under hypoxia condition. Three bio-replications were performed for each experiment. ***p<0.001.

(G) Intracellular ATP level was measured in MDA-MB-231 cells with DAB2IP overexpression (Myc-DAB2IP) and/or HIF-1α overexpression (Flag-HIF-1α) under hypoxia condition. Three bio-replications were performed for each experiment. **p<0.01; ***p<0.001.

(H) Lactate production level was measured in MDA-MB-231 cells with DAB2IP overexpression (Myc-DAB2IP) and/or HIF-1α overexpression (Flag-HIF-1α) under hypoxia condition. Three bio-replications were performed for each experiment. **p<0.01; ***p<0.001.

(I) Diagram of truncated forms of DAB2IP protein. FL: full length (1-1189); N: N-terminal segment (1-563); C: C-terminal segment (564-1189).

(J) Deletion mutants of DAB2IP C-terminal segment. C: C-terminal segment (564-1189); C-△PER: C-terminal segment with PER domain deletion (△592-718); C-△PR: C-terminal segment with PR domain deletion (△728-735); C-△LZ: C-terminal segment with LZ domain deletion (△843-860).

**S.Table 1. The siRNAs sequence and primers used in plasmid mutagenesis in this study.**

| **siRNAs** | **Target Sequence (5`-3`)** |
| --- | --- |
| siDAB2IP-1# | GGTGAAGGACTTCCTGACA |
| siDAB2IP-2# | GGAGCGCAACAGTTACCTG |
| siSTUB1-1# | CCCAAGTTCTGCTGTTGGACT |
| siSTUB1-2# | GAAGAGGAAGAAGCGAGACAT |
| siHIF1α-1# | CCGCTGGAGACACAATCATAT |
| siHIF1α-2# | CCAGTTATGATTGTGAAGTTA |
| shDAB2IP-1# | CCGGTTCGAGGTGACGACGTCATCACTCGAGTGATGACGTCGTC ACCTCGAATTTTTTG |
| shDAB2IP-2# | ACCGGTGGAGCGCAACAGTTACCTGTTCAAGAGCAGGTAACTGT TGCGCTCCTTTTTTGGTACCGAATTC |
| **Plasmid mutagenesis** | **Primers (5`-3`)** |
| C-ΔPER-Forward | TTTGTCACACGTGGCCTTGGCGACTCAGGCTCT |
| C-ΔPER-Reverse | AAGGCCACGTGTGACAAAAAACAAGTCCTTGTTT |
| C-ΔPR-Forward | TGCCACGGCAGAACCCTCCGCCCCCACCCCCGC |
| C-ΔPR-Reverse | GAGGGTTCTGCCGTGGCACCGTGGGCTGCCCGC |
| C-ΔLZ-Forward | GTAAGGACAAGAAGCTGGAGGAGTATGAGACCC |
| C-ΔLZ-Reverse | CCAGCTTCTTGTCCTTACTCCTTAGCCTATCCCTG |

**S.table 2. Antibodies used in the experiment.**

| **Antibodies** | **SOURCE** | **IDENTIFIER** |
| --- | --- | --- |
| Rabbit anti-DAB2IP | Abcam | Cat# ab87811, RRID:AB_2041032 |
| Rabbit anti-HIF1α | Cell signaling technology | Cat# 36169, RRID:AB_2799095 |
| Rabbit anti-STUB1 | Cell signaling technology | Cat# 2080, RRID:AB_2198052 |
| Rabbit anti-GLUT1 | Cell signaling technology | Cat# 12939, RRID:AB_2687899 |
| Rabbit Control IgG | Cell signaling technology | Cat# 3900, RRID:AB_1550038 |
| Rabbit anti-PGK1 | Thermo Fisher Scientific | Cat# PA5-28612, RRID:AB_2546088 |
| Mouse anti-Flag | Abclonal | Cat# AE005, RRID:AB_2770401 |
| Mouse anti-HA | Abclonal | Cat# AE008, RRID:AB_2770404 |
| Mouse anti-Myc | Abclonal | Cat# AE010, RRID:AB_2770408 |
| Rabbit anti-β-actin | Abclonal | Cat# AC038, RRID:AB_2863784 |
| HRP, Goat Anti-Rabbit IgG | Abbkine | Cat# A21020, RRID:AB_2876889 |
| HRP, Goat Anti-Mouse IgG | Abbkine | Cat# A21010, RRID:AB_2728771 |
